# Behavioral and metabolic risk factors associated with periodontitis in Brazil, 1990–2019: a multidimensional analysis for the Global Burden of Disease Study 2019

**DOI:** 10.1007/s00784-023-05384-6

**Published:** 2023-11-27

**Authors:** Silas Alves-Costa, Fábio Renato Manzolli Leite, Lorena Lúcia Costa Ladeira, Fernanda Lima-Soares, Antonio Marcus de Andrade Paes, Bruno Feres de Souza, Gustavo G. Nascimento, Cecilia Claudia Costa Ribeiro

**Affiliations:** 1 Postgraduate Program of Dentistry, Federal University of Maranhão, São Luís, MA Brazil; 2 National Dental Research Institute Singapore, National Dental Centre Singapore, Singapore, Singapore; 3 Duke-NUS Medical School, Oral Health ACP, Singapore, Singapore; 4 Department of Dentistry and Oral Health, Aarhus University, Aarhus, Denmark; 5 Postgraduate Program of Health Sciences, Federal University of Maranhão, São Luís, MA Brazil; 6 Postgraduate Program of Public Health, Federal University of Maranhão, São Luís, MA Brazil; 7 National Dental Research Institute Singapore, National Dental Centre Singapore, 5 Second Hospital Avenue, 168938 Singapore, Singapore

**Keywords:** Periodontitis, Behavioral risk factors, Metabolic risk factors, Noncommunicable diseases

## Abstract

**Objectives:**

Periodontitis is a non-communicable disease (NCD) that may be linked to other NCDs through shared risk factors. Accordingly, we analyzed the relationship between periodontitis and behavioral and metabolic risks common to NCDs in Brazilian adults over three decades.

**Methods:**

Indicators of periodontitis, behavioral risks (smoking, alcohol use, sugar-sweetened beverages (SSB), and physical activity), and metabolic risks (overweight/obesity, dyslipidemia, hyperglycemia, and hypertension) in Brazilian adults (25–49 y-old) between 1990 to 2019 were obtained from the Global Burden of Disease Study 2019. Data were adjusted for Gini index. Fixed-effects and Prais-Winsten regressions were performed (*p* < 0.05).

**Results:**

The prevalence of periodontitis has increased among Brazilians since 2005. High-SSB diet, alcohol use, and metabolic risks increased between 1990–2019, whereas smoking decreased. In crude models, periodontitis prevalence increased with alcohol use (2545.1; 95%CI: 2307.9–2782.3), high-SSB diet (365.5; 95%CI: 322.5–408.4), low physical activity (1784.4; 95%CI: 763.7–2805.0), overweight/obesity (172.3; 95%CI: 156.3–188.4), dyslipidemia (734.5; 95%CI: 624.7–844.2), and hyperglycemia (1774.3; 95%CI: 1555.9–1992.7). After adjustment for the Gini index, periodontitis prevalence raised with a high-SBB diet (1416.0; 95%CI: 1120.2–1711.8), overweight/obesity (629.9; 95%CI: 573.1–686.8), dyslipidemia (2035.8; 95%CI: 1728.1–2343.5), and hyperglycemia (8918.1; 95%CI: 7979.8–9856.3).

**Conclusions:**

Periodontitis has increased in Brazil since 2005, despite the smoking reduction. Sugar-sweetened beverage was the behavioral risk that mostly accompanied the periodontal trend.

**Clinical relevance:**

Our results support upstream strategies targeting commercial, social, political, and structural determinants to tackle NCDs and reduce oral health inequities.

**Supplementary information:**

The online version contains supplementary material available at 10.1007/s00784-023-05384-6.

## Introduction

Noncommunicable diseases (NCDs), such as cardiovascular disease, cancers, respiratory diseases, and diabetes, are responsible for approximately 70% of deaths worldwide, comprising 85% of premature deaths in low- and middle-income countries [1]. Periodontitis is a chronic disease manifested as irreversible inflammatory destruction of teeth apparatus and is one of the most prevalent NCDs [2]. Although not directly linked to mortality, periodontitis can predict other fatal NCDs decades earlier, such as diabetes and cardiovascular diseases [3].

The American Academy of Periodontology and the European Federation of Periodontology point out that periodontitis results from the interaction between a dysbiotic microbiome and the host-dysregulated immune response. Furthermore, they recognize that smoking and diabetes are risk factors that affect the severity, extent, and treatment response of periodontitis. In contrast, obesity, physical activity, and nutrition were noted as emerging risk factors that need future confirmation as etiological factors for periodontitis [4].

Systematic reviews have shown that alcohol consumption [5], sugar intake [6], and metabolic risk factors, e.g., obesity and hyperglycemia [7], are associated with an increased risk for periodontitis. In contrast, physical activity has been associated with an opposite trend [8]. However, to the best of our knowledge, no nationally representative sample studies have investigated all of those major behavioral and metabolic risk factors for NCDs associated with the rate of periodontitis from a longitudinal perspective.

Brazil is a continental country with peculiarities that make it suitable for assessing trends in behavioral and metabolic risk factors associated with periodontal disease. Brazil's smoking rate has markedly declined over the past few years [9], resulting from an efficient tobacco control policy implemented at the end of the eighties [10]. Meanwhile, the prevalence of periodontitis in Brazil has grown above the global mean in the last three decades [11]. Besides that, other behavioral risk factors, such as added sugar and alcohol consumption, have escalated in the country [12]; so, have metabolic risk factors, i.e., overweight/obesity, hyperglycemia, hypertension, and dyslipidemia rates [9]. Sifting through the literature, studies investigating the prevalence of periodontitis and the risk factors for NCDs after all these transformations are not found. For instance, even though tobacco smoking is an acknowledged cause of periodontitis [4] and its reduction is expected to decrease the burden of periodontitis in the population, it remains uncertain whether other behavioral and metabolic risk factors can nullify the effect of a decrease in exposure to tobacco.

Thus, we hypothesize that the prevalence of periodontitis has been increasing in Brazil over the last three decades, accompanied by added sugar and alcohol consumption and, consequently, overweight/obesity, hyperglycemia, hypertension, and dyslipidemia rates, despite the smoking reduction. Accordingly, we analyzed the prevalence of periodontitis over the last three decades (1990 to 2019) in Brazilian adults and estimated the association between the burden of behavioral and metabolic risk factors with periodontitis rate.

## Methods

### Study design

The present study uses panel data from 26 Brazilian states from adults (25 to 49 years) over three decades (1990 to 2019). The data were obtained from the Global Burden of Disease Study 2019 (GBD) [13] and the Brazilian Institute of Geography and Statistics (IBGE). The GBD systematically quantifies global disease and risk factor burdens across 204 countries and territories, enabling comparisons over time, among populations, and across health issues. The primary source of GBD data is national or state representative surveys, and it imputes missing data [14, 15]. Supplementary Table S1 contains descriptive information about the data herein used and its source.

#### Outcome

The periodontal disease provided by the GBD was the prevalence rate of periodontitis (per 100,000 inhabitants), defined in the study as a Community Periodontal Treatment Needs Index (CPITN) code IV, clinical attachment loss (CAL) > 6 mm, or probing pocket depth (PPD) > 5 mm [16].

#### Behavioral and metabolic variables

The main behavioral and metabolic risk factors for NCDs were chosen following the World Health Organization (WHO) risk factors for NDCs [1]. Accordingly, the behavioral risk factors were smoking, alcohol use, a diet high in sugar-sweetened beverages (SSB), and low physical activity. The metabolic risk factors were high body mass index (BMI) as an overweight/obesity marker, high LDL cholesterol as an indicator of dyslipidemia, high fasting plasma glucose as a hyperglycemia surrogate, and high systolic blood pressure as a proxy of hypertension. Due to their high collinearity, each independent variable was used in separate regression models. All these variables were available as the summary exposure value (SEV). The SEV measures a population’s exposure to a risk factor that considers the intensity of exposure, whose value varies from 0% (non-exposure) to 100% (fully-exposed).

The Gini index from 2005 to 2019 (Fig. S1), which measures the distribution of the Gross Domestic Product (GDP) representing income inequality, ranging from 0 (perfect equality) to 1 (maximum inequality), was used to adjust the models.

### Modeling approach

The models were fitted using fixed-effects modeling at a significance level of 0.05. State-level clustering was used to relax the autocorrelation and heteroskedasticity requirement [17]. Fixed-effect models are appropriate for assessing health outcomes because they can estimate unobservable location effects that remain fixed over time, i.e., geographic, and historical factors. Linear regressions were performed using R and RStudio.

The linear regression model consisted of a crude approach using the outcome explained by risk factors and the unobservable fixed-effect. A data matrix with 26 states × 30 years (t = 1990–2019) was used. The crude model was:

$${Periodontitis}_{it}={\beta }_{1}{RiskFactors}_{it}+{u}_{i}+{\varepsilon }_{it},$$

in which *Periodontitis* is the prevalence rate of periodontitis in a state *i* in year *t*. The *RiskFactors* are the SEV of the behavioral risk factors (i.e., smoking, alcohol use, a diet high in sugar-sweetened beverages, and low physical activity) and the metabolic risk factors (i.e., overweight/obesity, dyslipidemia, hyperglycemia, and hypertension). *u*_*i*_ is the unobservable location effect, and *ε*_*it*_ is an error term.

Adjusted analysis was performed using the Gini index. As in Brazil the Gini index has not been present since 1990, and evidence suggests a disturbance in the prevalence of periodontitis estimation in Brazil around the 2000s (Fig. S2) [18], thus, regression analyses were conducted using a data matrix with 26 states × 15 years (t = 2005–2019). The Gini index was chosen because it is the longest series available for the Brazilian states in panel data format and has already been associated with health indicators in the country [19]. Hence, the adjusted regression model was:

$${Periodontitis}_{it}={\beta }_{1}{RiskFactors}_{it}+{\beta }_{2}{Gini}_{it}+{u}_{i}+{\varepsilon }_{it}.$$

As a sensitivity analysis, we performed models for the two main behavioral risk factors, smoking, and a diet high in SSB, in the two intervals, 1990–2005 and 2005–2019.

Prais-Winsten estimations were performed to explore trends in time series of the prevalence of periodontitis and the summarized SEV of behavioral and metabolic risk factors. The GBD gives prevalence and SEV in summary form for Brazil, thus, the data matrix analyzed were 1 country × 30 years (19902019) and 1 country × 15 years (2005–2019). Results were classified as stationary (*p* > 0.05), increased (β_1_ > 0), or decreased (β_1_ < 0), given a *p* < 0.05. Logarithmic transformation was used to reduce the variance heterogeneity of the residuals. The annual percent change (APC) was also performed using the formula: 100*(10^β^ – 1), being β the coefficient of the Prais-Winsten regression [20].

#### Role of the funding source

This study was financed by the Coordination for the Improvement of Higher Education Personnel (CAPES). The funding agency did not influence the design, data collection, analysis, or publication decision. All authors had full access to the data and scientific independence to interpret them.

## Results

**Fig. 1:**
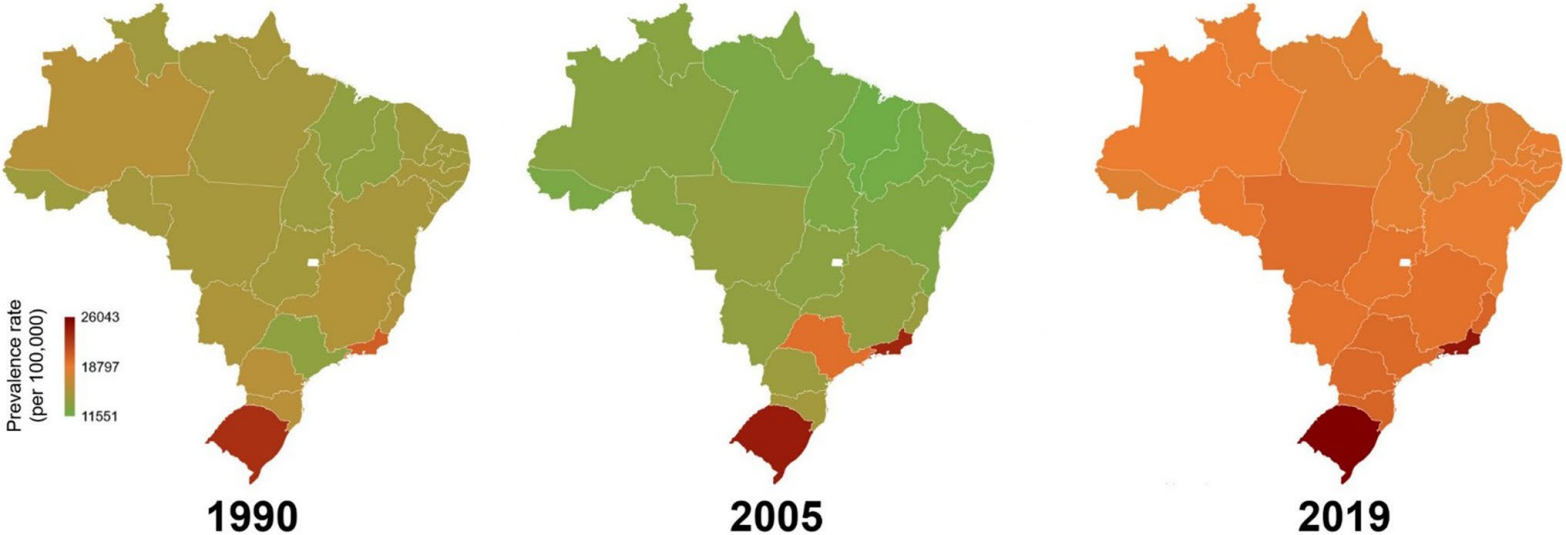
Prevalence rate of periodontitis in Brazilian states among adults (25 to 49 y-old), 1990, 2005, and 2019

The distribution of periodontitis in the Brazilian states among adults (25 to 49 years old) in 1990, 2005, and 2019 is depicted in Fig. 1. The prevalence of periodontitis significantly increased between 2005 to 2019 (APC: + 3.4%; 95%CI: 1.9–5.0) in Brazil. However, over the three decades, an uptrend but non-significant prevalence (APC: + 2.0%; 95%CI: -1.2–5.2) was observed. Rio de Janeiro and Rio Grande do Sul were the states that consistently showed the highest rates of periodontitis.

**Fig. 2:**
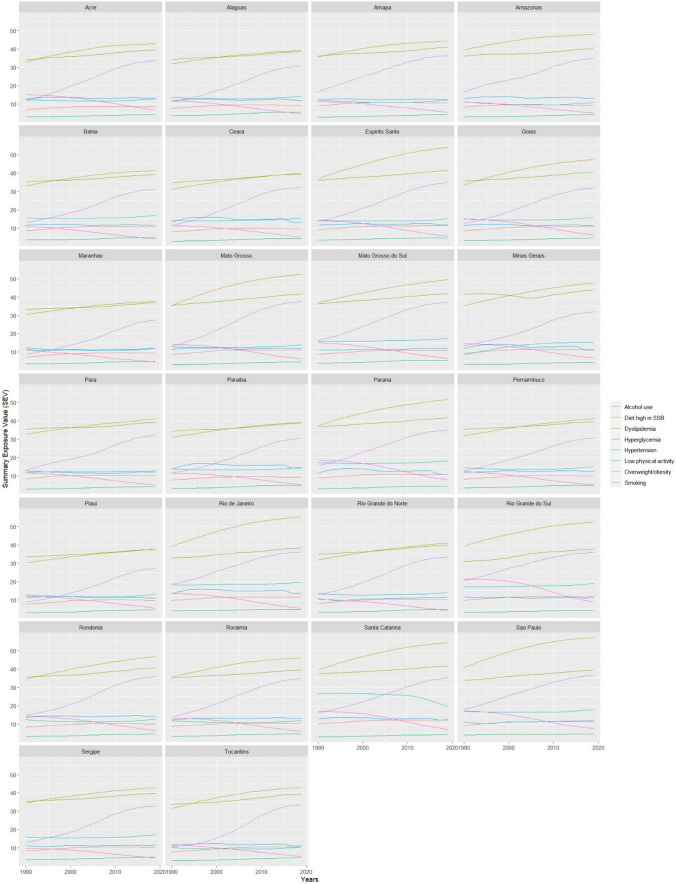
Summary exposure value of behavioral and metabolic risk factors in Brazilian states among adults (25 to 49 years) between 1990 and 2019

Table 1 shows the time series for each behavioral and metabolic risk factor in Brazil. There was an increase in the SEV of diet high in SSB (APC: + 2.4%; 95%CI: 1.9–2.8) and alcohol use (APC: + 1.5%; 95%CI: 0.9–2.2), in addition to the SEV of overweight/obesity (APC: + 9.9%; 95%CI: 8.7–9.3), dyslipidemia (APC: + 1.1%; 95%CI: 1.0–1.1) and hyperglycemia (APC: + 3.1%; 95%CI: 2.2–4.1) over the three decades. The SEV of smoking decreased from 14.9% in 1990 to 6.3% (APC: -6.7%; 95%CI: -7.8–-5.5). From 2005 to 2019, among the SEV of behavioral factors, only a diet high in SSB (APC: + 1.3%; 95%CI: 1.1–1.5) increased, whereas smoking decreased (APC: -9.5%; 95%CI: -9.7– -9.2). In this period, all SEV of metabolic risk factors increased in the country, mainly overweight/obesity (APC: + 4.6%; 95%CI: 3.5–5.7). Figure 2 displays the time series of behavioral and metabolic risk factors by state. An upward trajectory of SSB consumption was observed across all states, in contrast to smoking trends. Notably, the states of São Paulo, Rio de Janeiro, and Rio Grande do Sul exhibited the highest rates of SSB consumption in Brazil.

**Table 1:** Time series trend analysis of the prevalence of periodontitis and each SEV of behavioral and metabolic risk factors separately among Brazilian adults (25 to 49 years) from 1990–2019

	1990	2019	β_1_^a^	APC^b^	95%CI	T^c^	R^2^	2005	2019	β_1_^a^	APC^b^	95%CI	T^c^	R^2^
Periodontitis	15708.4	19942.2	0.008	2.0	-1.2–5.2	●	0.99	16233.4	19942.2	0.015*	3.4	1.9–5.0	▲	0.99
Behavioral risk factors														
Smoking	14.9	6.3	-0.030*	-6.7	-7.8– -5.5	▼	0.95	11.4	6.3	-0.043*	-9.5	-9.7– -9.2	▼	0.99
Alcohol use	9.0	10.9	0.007*	1.5	0.9–2.2	▲	0.97	10.9	10.9	< 0.001	< 0.1	-0.1–0.0	●	0.99
Diet high in SSB	36.7	49.2	0.010*	2.4	1.9–2.8	▲	0.99	45.5	49.2	0.006*	1.3	1.1–1.5	▲	0.99
Low physical activity	11.9	12.0	0.001	< 0.1	-0.6–0.6	●	0.99	12.2	12.0	-0.001	-0.3	-1.3–0.6	●	0.99
Metabolic risk factors														
Overweight/obesity	15.5	34.0	0.037*	9.9	8.7–9.3	▲	0.99	25.9	34.0	0.020*	4.6	3.5–5.7	▲	0.99
Dyslipidemia	35.1	40.1	0.005*	1.1	1.0–1.1	▲	0.99	37.3	40.1	0.005*	1.2	1.1–1.3	▲	0.99
Hyperglycemia	3.5	4.7	0.013*	3.1	2.2–4.1	▲	0.64	4.1	4.7	0.008*	2.0	1.5–2.4	▲	0.99
Hypertension	15.3	16.4	< 0.001*	-0.1	-0.2–0.0	▼	0.14	15.5	16.4	0.004*	0.9	0.9–0.5	▲	0.99

*Note:* *p < 0.05 for Prais-Winsten regression; ^a^β_1_- Obtained from Prais-Winsten regression; ^b^APC – Annual percent change (%); ^c^T – Trend in time series: ● (stationary), ▲ (increased), and ▼ (decreased). The values presented refer to the trend analysis of the time series for each variable separately

In the crude regression models, the SEV of behavioral risk factors alcohol use (365.5; 95%CI: 2307.9–2782.3), diet high in SSB (365.5; 95%CI: 322.5–408.4), and low physical activity (1784.4; 95%CI: 763.7–2805.0), and the metabolic risk factors overweight/obesity (172.3; 95%CI: 156.3–188.4), dyslipidemia (734.5; 95%CI: 624.7–844.2), and hyperglycemia (1774.3; 95%CI: 1555.9–1992.7) were positively associated with an increase in the prevalence of periodontitis in Brazil. Conversely, the SEV of smoking was negatively associated with periodontitis prevalence (-398.3; 95%CI: -473.6 – -323.0) over the study period. The linear regression coefficients are given in Table 2.

**Table 2:** Crude linear regression β-coefficients of the SEV of behavioral and metabolic risk factors for the prevalence rate (per 100,000 habitants) of periodontitis in the 26 Brazilian states (1990–2019)

	β-coefficient^a^	95% CI^b^	R^2^ within
Behavioral risk factors			
Smoking	-398.3*	-473.6– -323.0	0.0870
Alcohol use	2545.1*	2307.9–2782.3	0.2107
Diet high in SSB^c^	365.5*	322.5–408.4	0.1387
Low physical activity	1784.4*	763.7–2805.0	0.0534
Metabolic risk factors			
Overweight/obesity	172.3*	156.3–188.4	0.1068
Dyslipidemia	734.5*	624.7–844.2	0.1027
Hyperglycemia	1774.3*	1555.9–1992.7	0.0637
Hypertension	195.8	-421.5–813.3	0.0015

*Note:* *p < 0.05; ^a^β-coefficient – Obtained from fixed-effect regression; ^b^CI – Confidence Interval; ^c^SSB – sugar-sweetened beverages

Regarding the behavioral risk factors, when the models were adjusted for the Gini index (2005–2019), an increase in the prevalence rate of periodontitis was observed for each percentage increase in the SEV of a diet high in SBB (1416.0; 95%CI: 1120.2–1711.8). The SEV of smoking was negatively associated with the prevalence of periodontitis (-1103.9; 95%CI: -1433.2– -774.5). Concerning the metabolic risk factors, an increase in the prevalence rate of periodontitis accompanied the percentage increase in the SEV of overweight/obesity (629.9; 95%CI: 573.1–686.8), dyslipidemia (2035.8; 95%CI: 1728.1–2343.5), and hyperglycemia (8918.1; 95%CI: 7979.8–9856.3). The linear regression coefficients for behavioral and metabolic risk factors adjusted for the Gini index from 2005 to 2019 are described in Table 3. The sensitivity analysis for smoking and SSB consumption in the two intervals (1990–2005 and 2005–2019) yielded consistent results (Table S2).

**Table 3:** Gini index-adjusted linear regression β-coefficients of the SEV of behavioral and metabolic risk factors for the prevalence rate (per 100,000 habitants) of periodontitis in the 26 Brazilian states between 2005 and 2019

	β-coefficient^a^	95% CI^b^	R^2^ within
Behavioral risk factors			
Smoking	-1103.9*	-1433.2– -774.5	0.7145
Alcohol use	-6727.9	-13,754.4–298.5	0.1540
Diet high in SSB^c^	1416.0*	1120.2–1711.8	0.7460
Low physical activity	437.0	-644.3–1518.3	0.0998
Metabolic risk factors			
Overweight/obesity	629.9*	573.1–686.8	0.9208
Dyslipidemia	2035.8*	1728.1–2343.5	0.8286
Hyperglycemia	8918.1*	7979.8–9856.3	0.8787
Hypertension	1046.5	-1225.1–3318.1	0.1828

*Note:* *p < 0.05; ^a^β-coefficient – Obtained from fixed-effect regression; ^b^CI – Confidence Interval; ^c^SSB – sugar-sweetened beverages

## Discussion

Periodontitis rates have increased significantly among Brazilians since 2005, similar to increased consumption of sugar-sweetened beverages, overweight/obesity, dyslipidemia, and hyperglycemia. Furthermore, the prevalence of periodontitis rose despite the reduction of smoking. These findings reinforce the hypothesis that reducing smoking alone is not enough to reduce periodontitis burden. This study has the advantage of using the GBD database, providing complete and standardized data, and making it possible to compare population parameters over time in panel analysis.

The prevalence of periodontitis has been linked to the consumption of SSBs in Brazil. This observation may be endorsed by the highest rates of periodontitis in the states of Rio de Janeiro and Rio Grande do Sul, which also have consistently exhibited elevated levels of SSB consumption. Trend analyses showed that sugar-sweetened beverages' consumption rates increased significantly in Brazil over the three decades analyzed. These beverages are the primary source of discretionary calories in the Western diet. Sugar-sweetened beverages have been associated with metabolic risks such as obesity, insulin resistance, and hypertension [21]. Sustained insulin resistance leads the liver into *de novo lipogenesis* and consequent low-grade inflammation [22]. Increasing evidence points to the role of reactive oxygen species in establishing an oxidative stress environment that underlies the pathogenesis of periodontitis [23]. Periodontitis is highly prevalent in patients with type 2 diabetes, smoking habits, and obesity. Moreover, sugar consumption has been associated with cardiovascular risk [24] and also periodontal disease in adolescents [25] and young adults [26]. Poor dietary habits can worsen clinical periodontal parameters, contributing to oral dysbiosis. Sugars function as local stressors for the oral biofilm, which can create a highly proteic feeding environment for gram-negative bacterial species [27]. Inflammation-induced loss of periodontal tissue further exacerbates this situation. Other causative factors such as genetic factors and smoking may also contribute to periodontal lesion chronification, leading to a positive feedback loop that can worsen the deterioration of the tooth apparatus [28].

Our results from the crude analysis showed that an increase in alcohol consumption was associated with an increase in periodontitis burden over the last three decades, previous corroborating findings, including among Brazilians [29]. Alcohol use may reduce the immune response, favoring periodontitis development probably through maladaptive immunity [30]. Additionally, liver inflammation via acetaldehyde production may be observed, inducing *de novo lipogenesis,* and promoting hepatic insulin resistance, dyslipidemia, and hepatic steatosis, similar to the effects caused by added fructose [22]. Thus, alcohol use may result in or hasten low-grade systemic inflammation with oral consequences [22]. However, the alcohol use loss association after the model adjustment for the Gini index. Some hypotheses could explain this finding. First, the stationarity rate of alcohol use in the analyzed period (Table 1) could mask the association. Second, improving the Gini index (Fig. S1) in Brazil could mitigate the effects of alcohol consumption on periodontitis in the population since the alcohol harm paradox emphasizes that disadvantaged groups would be more exposed to alcohol-related problems [31]. Third, it is known that there is a consistent relationship between alcohol and periodontitis among males [5], whereas this study used an indicator for both sexes combined.

Similarly, low physical activity was also significantly associated in crude analysis with periodontitis prevalence. Physical activity could reduce the prevalence of periodontitis by reducing systemic inflammatory levels [8]. A study with obese individuals showed that weight loss induced by physical activity and a healthy diet might increase the expression of adiponectin receptors in adipose tissue and skeletal muscles [32]; an essential hormone regulator of insulin sensitivity and a biomarker for the risk of NCDs. However, the low physical activity rate in the Brazilian population did not account for the increase in periodontitis after adjustment for the Gini index. We suppose that the stationary level of this indicator between 2005 and 2019 was responsible for the loss of association after adjustment (Fig. 2 and Table 1).

Increased overweight/obesity also explained a greater periodontitis burden with the highest magnitude of association (R^2^ within = 0.920) among all the explored metabolic risk factors. In this sense, studies hypothesize that the white adipose tissue secretes adipocytokines, which, combined with the hypoxia caused by the expanded adipose tissue [33], could alter the innate immune response, increasing the susceptibility to inflammation and bacterial infections in periodontitis [34]. Since metabolic alterations are usually associated, hyperglycemia and dyslipidemia also explained the increase in periodontitis from 2005 to 2019 (R^2^ within > 0.820). A previous study has shown their association with periodontal disease [35]. Data suggest that the odds of periodontitis increase with the number of components of the metabolic syndrome present in an individual [35]. In fact, the progress of a hyperglycemic state together with a dyslipidemic profile can result in periodontal inflammation caused by systemic oxidative stress and a cascade of cytokines resulting from this process [23].

Surprisingly, our results demonstrate an inverse relationship, with periodontitis burden increasing despite reduced smoking rates. This unexpected outcome is likely linked to a substantial reduction in smoking (-58.14%) driven by Brazil's anti-smoking policies initiated in the 1980s [10]. The data suggests that targeting smoking reduction alone may not effectively mitigate the burden of periodontitis within the population, as it continued to rise. Therefore, we emphasize the necessity of proposing population-level policies addressing other behavioral and metabolic risk factors. In addition to the variables examined in this study, it is essential to contextualize the observed increase in periodontitis in Brazil during the study period. Various factors may have contributed to this trend, including dietary changes, such as the rise in ultra-processed foods [36, 37], limited access to dental care services, especially in underserved areas [38], as well as socio-economic and cultural factors.

Our findings support upstream strategies targeting commercial, social, political, and structural determinants to tackle NCDs and also reduce oral health inequities. For example, regulatory market policies, labeling, and taxation of products rich in added sugars in connection with subsidies to encourage a healthy diet including more fruits and vegetables can be efficient measures to tackle and prevent NDCs, as learned from the smoking case, whose reduction was associated with a lower periodontitis burden.

As limitations, our findings may be affected by underreporting some indicators since we used secondary data. The primary data source for the GBD information is national or state surveys and missing data are estimated using a smoothing algorithm designed for the database. Additionally, the use of ecological data from Brazilian states for linear regression may not allow for individual-level interpretations due to potential ecological fallacy. Given the close relationship of the conditions under study (periodontitis, behavioral, and metabolic risk factors) to socioeconomic inequalities, our aggregate-level data provide average estimates for both poor and wealthy individuals, requiring cautious interpretation. Despite nearly 30 years of data, our approach may not fully capture the chronic nature of these conditions, particularly periodontitis. The influence of risk factors may require a time lag to manifest, necessitating longer analysis periods to assess cumulative effects fully. Lastly, causal inferences should not be drawn from our study, as despite using fixed effects and control variables, the presence of unobserved time-varying confounding factors cannot be entirely ruled out.

The rate of periodontitis has been increasing in Brazil since 2005, despite the smoking reduction. Sugar-sweetened beverage consumption was the behavioral risk factor that best explained this trend in Brazilians. This behavior is parallelly related to the increased accumulation of exposures to metabolic alterations, thus nullifying the beneficial effect of tobacco reduction. Therefore, interventions on upstream determinants of NDCs are of utmost importance to control metabolic and behavioral risk factors that influence periodontitis rates.

### Supplementary information

Below is the link to the electronic supplementary material.

Supplementary file1 (DOCX 5658 KB)

## Data Availability

The data utilized in this study is publicly available and can be freely accessed for download from https://vizhub.healthdata.org/gbd-results/ and https://sidra.ibge.gov.br/tabela/5939.
