# Supplementary material for: Behavioral and metabolic risk factors associated with periodontitis in Brazil, 1990–2019: a multidimensional analysis for the Global Burden of Disease Study 2019
Source: Clin Oral Investig. 2023 Nov 27;27(12):7909–17. doi: 10.1007/s00784-023-05384-6 (PMC10713794; doi:10.1007/s00784-023-05384-6)
Supplement: Supplementary file 1 — Supplementary file1 (DOCX 5658 KB) [file 784_2023_5384_MOESM1_ESM.docx]

**Supplementary material**

**Journal:** Oral Clinical Investigations

**Title:** Behavioral and metabolic risk factors associated with periodontitis in Brazil, 1990-2019: a multidimensional analysis for the Global Burden of Disease Study 2019.

**Authors:** Silas Alves-Costa; Fábio Renato Manzolli Leite; Lorena Lúcia Costa Ladeira; Fernanda Lima-Soares; Antonio Marcus de Andrade Paes; Bruno Feres de Souza; Gustavo G. Nascimento; Cecilia Claudia Costa Ribeiro

**Corresponding Author:**

Centre Singapore, National Dental Research Institute Singapore

5 Second Hospital Avenue, Singapore 168938, Singapore.

ggn@duke-nus.edu.sg

| **Variable name** | **Input data** | **Case definition** | **Measure** | **Source** |
| --- | --- | --- | --- | --- |
| Periodontitis | Periodontal diseases | Community Index of Periodontal Treatment Needs (CPITN) code IV, clinical attachment loss (CAL) > 6 mm, or periodontal pocket depth (PPD) >5mm. | Prevalence rate (per 100,000 habitants) | Institute for Health Metrics and Evaluation/ Global Health Data Exchange  Available at: https://vizhub.healthdata.org/gbd-results/ |
| Smoking | Smoking | Current daily or occasional use of any smoked tobacco product. | Summary Exposure Value (SEV) - A measure of a population’s exposure to a risk factor that takes into account the extent of exposure by risk level and the severity of that risk’s contribution to disease burden. SEV takes the value zero when no excess risk for a population exists and the value one when the population is at the highest level of risk; we report SEV on a scale from 0% to 100% to emphasize that it is risk-weighted prevalence. |  |
| Alcohol use | Alcohol use | Current drinkers as individuals consuming at least one alcoholic beverage in the past year. Among current drinkers, was estimated the level of exposure based on average grams of pure alcohol consumed per day. |  |  |
| Diet high in sugar-sweetened beverages | Diet high in sugar-sweetened beverages | Any intake (in grams per day) of beverages with ≥50 kcal per 226 .8 gram serving, including carbonated beverages, sodas, energy drinks, and fruit drinks, but excluding 100% fruit and vegetable juices. |  |  |
| Low physical activity | Low physical activity | It was measured in total metabolic equivalents (METs) and was defined as average weekly physical activity (at work, home, transport-related, and recreational) of less than 3000–4500 MET minutes per week. |  |  |
| Overweight/obesity | High body-mass index | It was defined as body-mass index (BMI) greater than 20–25 kg/m^2^. |  |  |
| Dyslipidemia | High LDL cholesterol | It was obtained estimating blood concentration of low-density lipoprotein (LDL) greater than 0 .7 and 1 .3 mmol/L. |  |  |
| Hyperglycemia | High fasting plasma glucose | It was defined as serum fasting plasma glucose of greater than 4 .8–5 .4 mmol/L. |  |  |
| Hypertension | High systolic blood pressure | It was defined as systolic blood pressure above 110-115 mm Hg |  |  |
| Gini index | Gini index | Gini index of gross domestic product at current prices - Reference year 2010. | Gini index - Gini index measures the extent to which the distribution of income (or, in some cases, consumption expenditure) among individuals or households within an economy deviates from a perfectly equal distribution. A Lorenz curve plots the cumulative percentages of total income received against the cumulative number of recipients, starting with the poorest individual or household. The Gini index measures the area between the Lorenz curve and a hypothetical line of absolute equality, expressed as a percentage of the maximum area under the line. Thus, a Gini index of 0 represents perfect equality, while an index of 1 implies perfect inequality. | Brazilian Institute of Geography and Statistics/ IBGE System of Automatic Recovery  Available at: https://sidra.ibge.gov.br/tabela/5939 |

**Table S1.** Variables and data sources descriptions used in the analysis^a^.

^a^ All data were obtained in a summarized form for Brazil and detailed for each Brazilian state, excluding the Federal District.

**
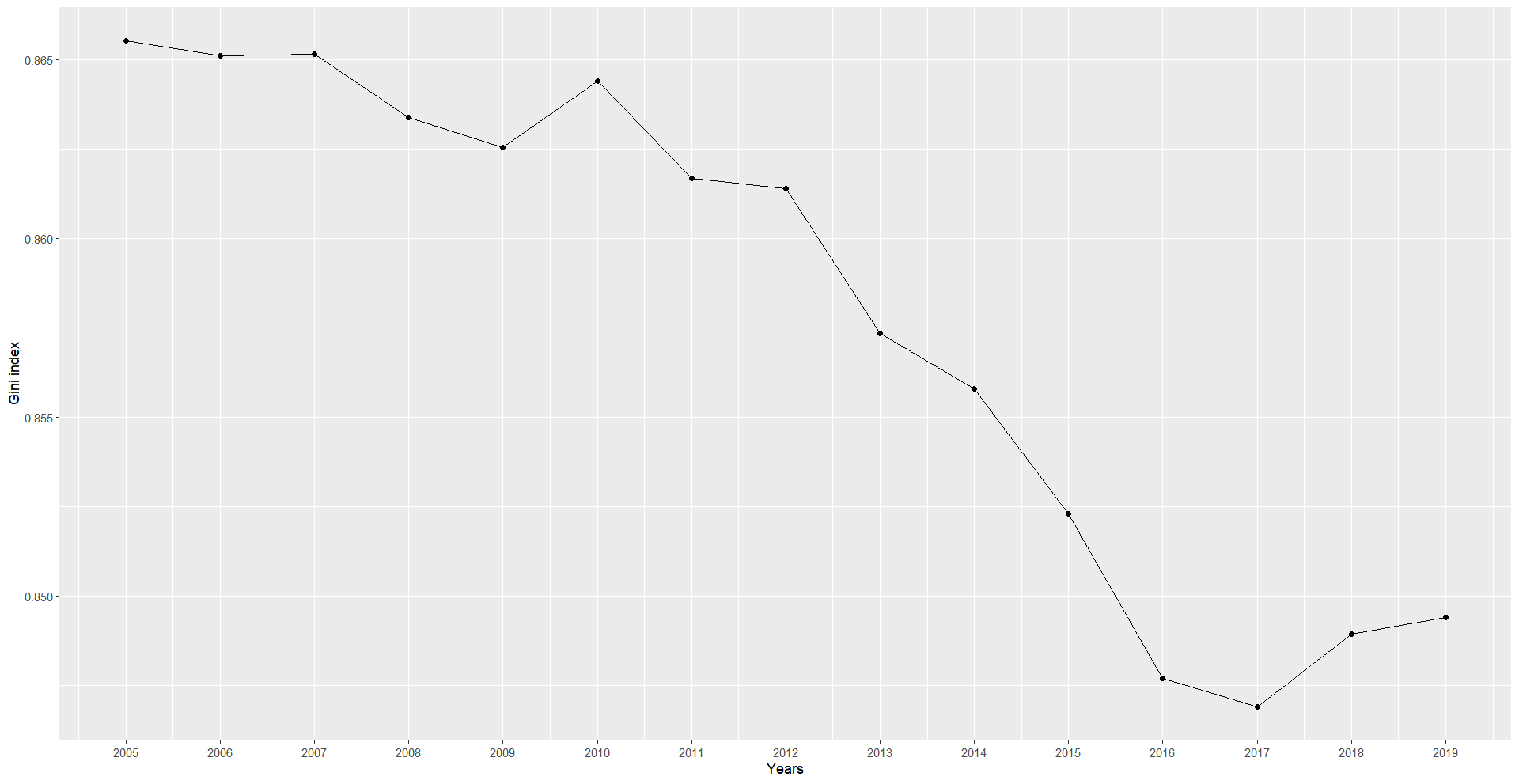
 Fig. S1** Gini index of the Gross Domestic Product distribution in Brazil (2005-2019)

**
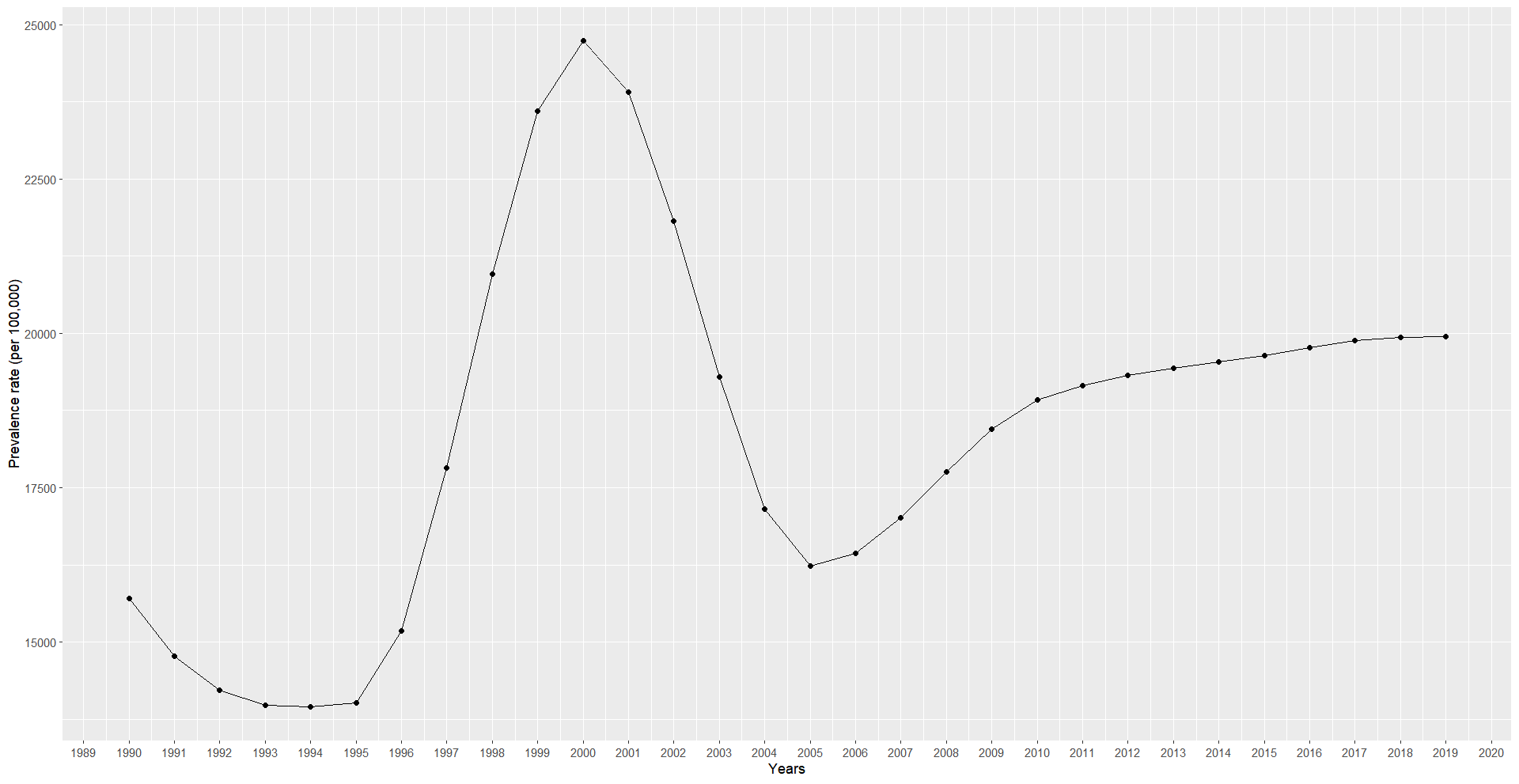
 Fig. S2** Prevalence rate (per 100,000) of periodontitis among Brazilian adults (25 to 49 years old) between 1990 to 2019

**Table S2.** Crude linear regression β-coefficients of the SEV of smoking and SSB consumption for the prevalence rate (per 100,000 habitants) of periodontitis in the 26 Brazilian states during two distinct time periods (1990-2005 and 2005-2019).

|  | **1990-2005** | |  | **2005-2019** | |
| --- | --- | --- | --- | --- | --- |
|  | **β-coefficient**^a^ | **SE**^b^ |  | **β-coefficient**^a^ | **SE**^b^ |
|  |  |  |  |  |  |
| Smoking | -1773.46* | 80.09 |  | -1072.21* | 157.56 |
| Diet high in SSB^c^ | 870.94* | 50.22 |  | 1408.19* | 146.19 |

*Note:* *p<0.05; ^a^β-coefficient – Obtained from fixed-effect regression; ^b^SE – Standardized Error; ^c^SSB - sugar-sweetened beverages.
